# Role of TGF-β/Smad Pathway in the Transcription of Pancreas-Specific Genes During Beta Cell Differentiation

**DOI:** 10.3389/fcell.2019.00351

**Published:** 2019-12-20

**Authors:** Yuhua Gao, Ranxi Zhang, Shanshan Dai, Xue Zhang, Xiangchen Li, Chunyu Bai

**Affiliations:** ^1^Institute of Precision Medicine, Jining Medical University, Jining, China; ^2^Institute of Animal Sciences, Chinese Academy of Agricultural Sciences, Beijing, China; ^3^Department of Spine Surgery, Qingdao Municipal Hospital, Qingdao, China; ^4^College of Animal Science and Technology, College of Veterinary Medicine, Zhejiang A&F University, Lin’an, China

**Keywords:** pancreatic beta cells, stem cells, TGF-β/Smad pathway, Ngn3, microRNAs

## Abstract

Autoimmune destruction of pancreatic beta cells causes absolute insulin deficiency and results in type 1 diabetes mellitus (T1DM). The substitution of healthy pancreatic beta cells for damaged cells would be the ideal treatment for T1DM; thus, the generation of pancreatic beta cells from adult stem cells represents an attractive avenue for research. In this study, a cocktail of factors was used to induce the differentiation of pancreatic beta cells from mesenchymal stem cells (MSCs). The differentiation program was divided into five stages, and the roles of the cocktail factors used during each stage were systematically elucidated. Activin A was found to phosphorylate Smad2 and Smad3 in stage III, thereby activating the TGF-β/Smad pathway. Meanwhile, the endocrine-specific transcription factor, Ngn3, and the pancreas-specific miRNAs, miR-375 and miR-26a, were dramatically elevated in stage III. We next demonstrated that Smad4, an important transcription factor in the TGF-β/Smad pathway, could bind to the promoter sequences of target genes and enhance their transcription to initiate the differentiation of beta cells. Use of SB-431542, an inhibitor of the TGF-β/Smad pathway, demonstrated *in vivo* and *in vitro* that this pathway plays a critical role in the production of pancreatic beta cells and in modulating insulin secretion. Thus, the TGF-β/Smad pathway is involved in the production of beta cells from adult stem cells by enhancing the transcription of Ngn3, miR-375, and miR-26a. These findings further underline the significant promise of cell transplant therapies for type 1 diabetes mellitus.

## Introduction

Pancreatic beta cells regulate blood glucose homeostasis through their production of insulin. Type 1 diabetes mellitus (T1DM) results from the autoimmune destruction of pancreatic beta cells. The replacement of these beta cells with healthy cells would be the ideal therapy for T1DM; thus, the production of pancreatic beta cells from adult stem cells or embryonic stem cells offers a promising avenue for research. Mesenchymal stem cells (MSCs) are attractive donor cells for cell transplantation, as they are multipotent and exert a strong immunoregulatory effect. Umbilical cord tissue is readily obtained from discarded term placentae without any risk to the donors. Umbilical cord MSCs are typical adult MSCs but with the added advantages of being easy to culture *in vitro* and of simpler ethical access compared with other stem cells. Therefore, umbilical cord MSCs are a promising candidate for cell therapy.

Genome-encoded microRNAs (miRNAs) regulate gene expression post-transcriptionally. These non-coding small RNAs (18–25 nt) regulate gene expression through binding to the 3′-untranslated regions of specific mRNAs and inhibiting their translation. The role of miRNAs in the regulation of beta cell differentiation has been demonstrated by the generation of a mouse model with beta cell-specific ablation of Dicer1 (Plaisance et al., 2014; Bai et al., 2016), and disruption of *Dicer1* in rats with the use of a insulin promoter 2 (RIP)-Cre transgene results in changed islet morphology, reduced pancreatic beta cell numbers, and dysregulated glucose-induced insulin secretion (GSIS) (Kalis et al., 2011). Many miRNAs have been shown to be important regulators in the differentiation and function of pancreatic beta cells, including let-7 (Krek et al., 2005; Lovis et al., 2008), miR-223, miR-21 (Du Rieu et al., 2010; Bai et al., 2016), miR-200, miR-30d, miR-124a (Tang et al., 2009), miR-26 (Bai et al., 2017a), miR-24, miR-148 (Melkman-Zehavi et al., 2011), miR-204 (Roldo et al., 2006), and miR-375 (Poy et al., 2004), as well as miR-146a, miR-15a, miR-29a, miR-9, miR-16, and miR-34 (Rosero et al., 2010; Bai et al., 2017b). However, as yet, there have been no reports regarding the role of induction factors in promoting the transcription of pancreatic miRNAs during beta cell differentiation from stem cells, and the molecular mechanisms underlying this process remain unclear.

The TGF-β superfamily of secreted polypeptide growth factors plays an important role in a variety of pathophysiologic processes, including vascular remodeling, angiogenesis, and atherogenesis, as well as in regulating cellular responses such as differentiation, proliferation, growth, adhesion, migration, survival, and the specification of developmental fate. Apart from TGF-β, this superfamily also includes the activins and the BMPs (bone morphogenetic proteins). Activins are dimeric proteins composed of either two βA subunits (activin A), two βB subunits (activin B) or a βA and βB subunit (activin AB). Activin A is extensively involved in the production of beta cells from stem cells (Shi et al., 2005; Pagliuca et al., 2014; Bai et al., 2017a) but the functions of the TGF-β pathway in beta cell differentiation and pancreatic miRNA expression have not been fully investigated.

In this study, we used a segmented induction method to produce beta cells from mouse umbilical cord MSCs, and we detected the expression of pancreatic miRNAs and the activation of the TGF-β/Smad pathway by examining quantitative reverse transcription PCR (RT-qPCR) and western blotting results of each stage of beta cell production. Combining our data with those from previous reports, we found that the pancreatic miRNAs, miR-26a and miR-375, play an important role in the formation of beta cells and in their secretion of insulin (Bai et al., 2017a, b), and that the TGF-β/Smad pathway plays an important role in regulating the transcription of these pancreatic miRNAs. To elucidate the mechanisms of transcriptional regulation during the production of beta cells and to better understand the interaction of the TGF-β/Smad pathway with pancreatic miRNAs expressed during the differentiation of beta cells from mouse umbilical cord MSCs, we tested both the activation and suppression of this pathway *in vitro*. The results confirmed the critical role of the TGF-β/Smad pathway during pancreatic beta cell differentiation.

## Materials and Methods

### Ethics Statement

This study was approved by the Committee on the Ethics of Animal Experiments of Jining Medical University (License ID: 2017-JZ-003). All surgery was performed under pentobarbital anesthesia, and all efforts were made to minimize suffering.

### Umbilical Cord MSC Culture and Differentiation Into Beta Cells

Wharton’s Jelly was selected from 16-old-day mice embryos and digested with collagenase type IV (Sigma-Aldrich, MO, United States) under sterile conditions to isolate the umbilical cord MSCs. Umbilical cord MSCs were cultured in complete medium containing L-DMEM (Gibco, Carlsbad, CA, United States), 10% FBS (Gibco), 100 mg/mL streptomycin, and 100 U/mL penicillin. Positive markers of MSCs, CD44 (1:500; ab157107, Abcam, Cambridge, MA, United States), CD90 (1:800; ab3105, Abcam), CD105 (1:500; ab107595, Abcam), and negative markers of MSCs, Ngn3 (1:500; ab216885, Abcam) and Pdx1 (1:800; ab47267, Abcam) were analyzed using flow cytometry (Bai et al., 2017b).

For the production of beta cells, we used the classical cocktail factor method, and the cocktail factors included 1 μM 5-aza-2′-deoxycytidine (5-AZA; Sigma-Aldrich), 0.1 mM β-mercaptoethanol (Sigma-Aldrich), 1 mM non-essential amino acids (Gibco), 15 ng/mL activin A (Sigma-Aldrich), 10 mM *all-trans* retinoic acid (ATRA; Sigma-Aldrich), 1% B27 (Gibco), 10 ng/mL bFGF (Peprotech, Rocky Hill, TX, United States), 10 mM nicotinamide, and 1% ITS (Gibco). In brief, umbilical cord MSCs were exposed to 1 μM 5-AZA for 18 h before induction. The cells were then cultured in low-glucose DMEM containing 10% FBS, 0.1 mM β-mercaptoethanol, and 1 mM non-essential amino acids. On day 5, the medium was supplemented with 15 ng/mL activin A. On day 7, 10 mM ATRA was added. On day 9, the medium was replaced with fresh medium supplemented with 1% B27, 10 ng/mL bFGF, 10 mM nicotinamide, and 1% ITS to encourage further differentiation (Bai et al., 2017a). On day 16, the cocktail factors were used to induce MSCs to achieve terminal beta cell differentiation, and the culture media was then replaced with beta cell media (DMEM medium containing 25 mM glucose, 70 μM β-mercaptoethanol, and 4 mM L-glutamine, and supplemented with 15% FBS).

### Glucose-Stimulated Insulin Secretion (GSIS)

GSIS was tested according to a previously reported procedure (Bai et al., 2016). Briefly, beta cells derived from MSCs were washed with Krebs buffer and then pre-incubated in Krebs buffer containing 2.5 mM glucose for 2 h to remove residual insulin. The cells were washed three times using Krebs buffer, incubated again in 2.5 mM glucose-Krebs buffer for 30 min, and the supernatant was selected. Next, the cells were washed three times and incubated in 20.5 mM glucose-Krebs buffer for 30 min, after which the supernatant was selected. This experiment was repeated four times. Finally, cell clusters were incubated in Krebs buffer containing 2.5 mM glucose. Cell supernatants containing secreted insulin were analyzed with an insulin ELISA test kit (EZRMI-13K; Millipore, Billerica, MA, United States) (Bai et al., 2017b).

### Flow Cytometry (FCM)

To analyze the expression of specific markers in the umbilical cord MSCs and pancreatic beta cells, these cells were analyzed using a Beckman Coulter FC500 flow cytometer (Beckman Coulter, Brea, CA, United States). Briefly, cells were selected using 0.125% (w/v) trypsin, separated into 500 μL aliquots, and labeled with FITC- or Cy5-conjugated antibodies against CD44 (1:500; ab157107, Abcam), CD90 (1:800; ab3105, Abcam), CD105 (1:500; ab107595, Abcam), Ngn3 (1:500; ab216885, Abcam), and Pdx1 (1:800; ab47267, Abcam) as per the manufacturer’s instructions (Hackstein et al., 2003). The FCM data were analyzed with CXP software (Beckman Coulter). Mean fluorescence intensity was analyzed after subtraction of the negative control signal.

### Immunofluorescence Detection

The MSCs or differentiated beta cells were fixed with 4% PFA (paraformaldehyde) for 20 min at room temperature and then incubated in 0.15% Triton X-100 for 10 min at room temperature. Next, PBS containing 4% (w/v) goat serum was applied for 30 min at room temperature or 2 h at 4°C. The cells were then incubated with the primary antibody overnight at 4°C. The FITC- or Cy5-labeled secondary antibody (Bioss, Beijing, China) was applied at a concentration of 10 μg/mL. Primary antibodies included anti-Ngn 3 (1:100; ab216885, Abcam) and anti-Pdx1 (1:1000; ab47267, Abcam). Photomicrographs were taken with a Nikon TE2000 confocal microscope with an attached Nikon ZE-1-C1 digital camera system (Nikon, Tokyo, Japan).

### Western Blotting

Ngn3, Pdx1, the Smads, Sox6, Bhlhe22, Mtpn, and Gapdh were analyzed using western blotting following the activation or suppression of the TGF-β/Smad pathway in umbilical cord MSCs. Cells were lysed using the Protein Extraction Reagent (Beyotime, Beijing, China) supplemented with a PI (protease inhibitor, Beyotime) to obtain whole-cell lysates. Nuclear extracts from umbilical cord MSCs were prepared as described previously using the Nuclear and Cytoplasmic Protein Extraction Kit (Beyotime) (Bai et al., 2019). Protein concentrations of the extracts were measured with the BCA assay (Beyotime) and equalized with extraction reagent. Extracts of equivalent total protein content were loaded and subjected to SDS-PAGE, followed by transfer onto 0.2 μm nitrocellulose membranes. Primary antibodies against Ngn3 (1:500); Pdx1 (1:1,000); p-Smad2 (1:400; 18338, Cell Signaling Technology, Danvers, MA, United States); p-Smad3 (1:500; 9520, Cell Signaling Technology); Smad2/3 (1:1,000; 8685, Cell Signaling Technology); Smad4 (1:500; 46535, Cell Signaling Technology); Sox6 (1:1,000; ab30455, Abcam); Bhlhe22 (1:2,000; ab204791, Abcam); Mtpn (1:1,000; ab241982, Abcam); Gapdh (1:5,000; ab181602, Abcam); and Histone (1:2,000; ab1791, Abcam) were used together with HRP (horseradish peroxidase)-coupled secondary antibodies (1:3,000; A0216 and A0208, Beyotime). Signals were developed using ECL (enhanced chemiluminescence) detection reagents on the nitrocellulose membranes. Gapdh and Histone were used as internal controls. Protein abundance was calculated and analyzed with ImageJ tools.

### MiRNA Quantitative Reverse-Transcription PCR (RT-qPCR)

MiRNA RT-qPCR was used to analyze the expression of pancreatic miRNAs during the differentiation of beta cells from umbilical cord MSCs. Twenty-two miRNAs, known to be involved in either pancreatic development and/or the maintenance of the function of beta cells, were selected for analysis after induction. These were miR-26a, miR-375, miR-100, miR-99, miR-125b, miR-181a, miR-92, miR-30, miR-221, miR-21, miR-19b, miR-33-5p, miR-142-3p, miR-30d, miR-33-3p, miR-27, miR-142-5p, miR-429, miR-29a, miR-222, miR-200, miR-128, miR-204, miR-223, miR-146a, miR-15, miR-21, miR-34, and miR-212/132. The miRNAs were obtained from umbilical cord MSCs and induced MSCs with the miRcute miRNA Isolation Kit V1.0 (Tiangen, Beijing, China), and the isolated miRNAs were then poly(A)-tailed and reverse-transcribed with the miRcute miRNA complementary DNA (cDNA) kit v2.0 (Tiangen). Quantitative PCR of these miRNAs cDNA was performed with the SYBR Green method using the miRcute miRNA quantitative PCR Detection Kit v2.0 (Tiangen) on the Roche Light Cycler 480 system and under the following conditions: 95°C for 15 min; 5 cycles at 94°C for 20 s, 65°C for 30 s, and 72°C for 34 s; 40 cycles at 94°C for 20 s, and 60°C for 34 s. The small nuclear RNA, U6, was used as an internal control for normalization. All miRNA primers were purchased from the miRNA primer bank of Tangen Biotech (Beijing, China). Each experiment was performed in duplicate and repeated three times. The relative expression of the miRNAs was calculated with the comparative threshold cycle (CT) method as 2^–ΔΔCT^. Changes in miRNA expression were illustrated with a heat map prepared using Mev-tm4^1^ and assessed with the *t*-test. A value for *p* < 0.05 was considered statistically significant.

### *In situ* PLA

Umbilical cord MSCs and induced MSCs were fixed with pre-chilled 4% PFA for 15 min. The cells were washed three times with PBS and then permeabilized with 0.15% (v/v) Triton X-100 for 10 min at room temperature. The cells were blocked for 0.5 h at room temperature or for 2 h at 4°C with 4% (w/v) bovine serum albumin (BSA; Gibco) containing 0.1% (v/v) Tween 20, and incubated with the antibody pairs (Smad 3 and Smad 4, or Smad 2 and Smad 4) for 1 h at 37°C. Duolink *In situ* PLA (Sigma-Aldrich) was performed according to previous reports (Bai et al., 2019). Images were scanned with a Nikon TE2000-E inverted confocal microscope (Nikon, Yokohama, Japan).

### ChIP-PCR Assessment

ChIP was executed with the ChIP assay kit (Beyotime) using the Smad 4 antibody, Smad 4, and anti-IgG antibodies (Cell Signaling Technology). The ChIP DNA was extracted with the DNA Purification Kit (Beyotime) and the samples were then subjected to qPCR amplification with primers spanning the protein-binding sites. The primers used were for miR-26a (F: 5′-GAGGCCTGATGGAGCCGTGGGGACC-3′, R: 5′-AGAG CCACAGCAGGCGGAAAGCCAGATGCCACAGG-3′), miR-375 (F: 5′-TTTCCTAGAACTGCTGTCTTGTCCCATCGCC CACA-3′, R: 5′-AAGAACCCACCCTTTCCTCATCAGAACCAC TTTGC-3′), and Ngn3 (F: 5′-TTAGGCTTTGGATTCTTCATAG TTCTGATAGGATTTGC-3′, R: 5′-AAGTGTCTTCTGGTCCC AGGAATGGAGGCGTAAGG-3′).

### EMSA (Electrophoretic Mobility Shift Assay)

Nuclei from induced MSCs were obtained according to previous reports (Bai et al., 2019) using the Nuclear and Cytoplasmic Protein Extraction Kit (Beyotime). The protein concentrations were tested using the bicinchoninic acid method. The probes used for EMSA are shown in Supplementary Table S1. Gel-shift assays were executed using the EMSA/Gel-Shift Kit (Beyotime). The supershifting antibody against Smad4 (46535, ChIP Grade) was purchased from Cell Signaling Technology.

### Transplantation *in vivo*

Transplantation *in vivo* was executed as previously described (Shi et al., 2005; Bai et al., 2017a). All animal procedures were approved by the Institutional Animal Care and Use Committee of the Jining Medical University. Streptozocin (STZ, optimal concentration of 50 mg/kg) was intraperitoneally injected each day for 5 days into 100 mice at 6 weeks of age. The Blood glucose of mice were measured from snipped tails every day after injection by GlucoTREND (Roche), the hyperglycemia (> 13.9 mM) was found after 7 days, and stabilized after 10 days. When the blood glucose levels of the mice rose above 13.9 mM, either 1 × 10^6^ normal MSCs, induced MSCs or SB-431542-treated induced MSCs were seeded into the renal capsule of the mice. For the sham control, cells were replaced with PBS. The blood glucose in the peripheral blood was measured every 4 days after transplantation.

### Statistical Analysis

All experiments were performed at least three times independently and repeated in triplicate. All data were analyzed using SPSS 16.0. All results represent the mean ± SD (standard deviation). The differences in the data were assessed with the Student’s *t*-test. *P* < 0.05 were considered statistically significant.

## Results

### Cocktail Factors Induce the Differentiation of Beta Cells From Umbilical Cord MSCs

**FIGURE 1:**
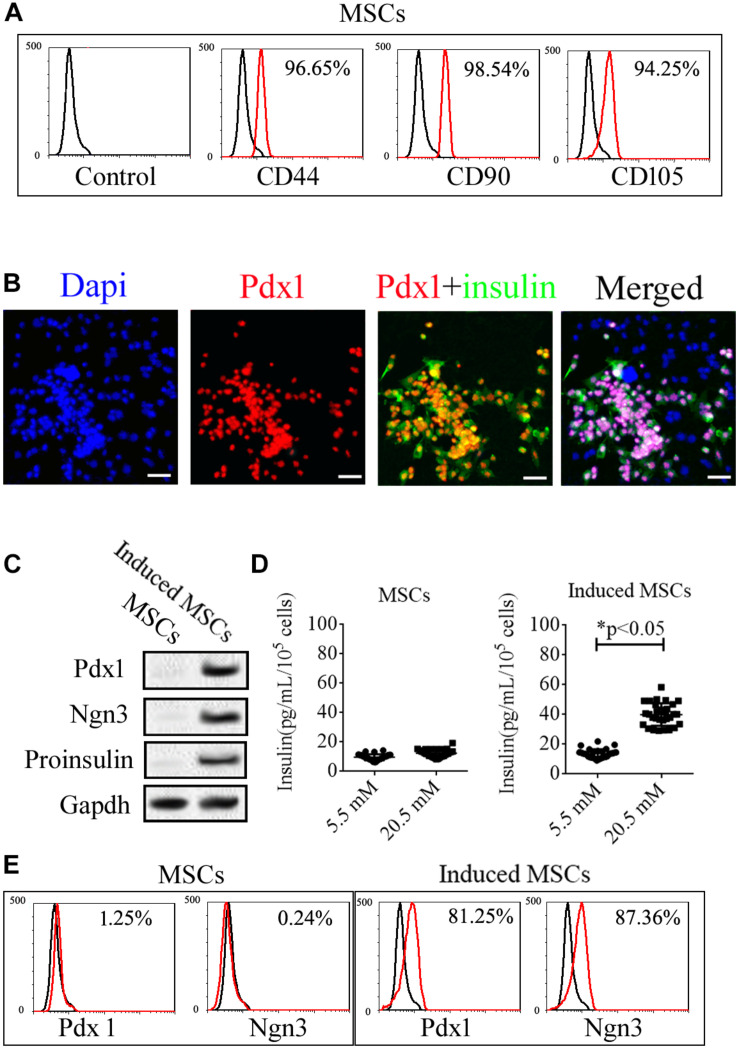
Expression of specific markers in uninduced and induced mice umbilical cord MSCs. **(A)** The isolated MSCs were positive for the MSC surface markers, CD44, CD90, and CD105, based on flow cytometry. **(B)** Mice umbilical cord MSCs were induced to differentiate into pancreatic beta cells with the use of a cocktail of factors, following which, the expression of the islet hormones, Pdx1 and insulin, was confirmed with immunofluorescence microscopy. **(C)** Based on western blotting, the induced MSCs exhibited expression of the islet hormones, Ngn3, Pdx1, and insulin, while the uninduced MSCs showed no expression. **(D)** Glucose-induced insulin secretion (GSIS) as measured by ELISA indicated that the insulin secretion of induced MSCs but not uninduced MSCs increased when treated with increasing glucose concentrations (*n* = 20; paired two-tailed *t*-test). **(E)** Expression of the islet markers, Pdx1 and Ngn3, in the induced MSCs was confirmed by flow cytometry.

Mice umbilical cord MSCs expanded rapidly to exhibit classical “fusiform” morphology after isolation from Wharton’s Jelly. The expression of specific markers in these cells, including CD44, CD90, CD105, Ngn3, and Pdx1, was then analyzed (Figure 1). Based on flow cytometry (FCM), the mice umbilical cord MSCs were positive for specific markers of MSCs including CD44, CD90, and CD105 (Figure 1A) but negative for specific markers of pancreatic beta cells including insulin, Ngn3, and Pdx1 based on immunofluorescence assays and western blotting (Figures 1B,C). We then used cocktail factors to induce the differentiation of beta cells from umbilical cord MSCs. Immunofluorescence microscopy and western blotting confirmed the expression of the islet hormones, Ngn3, Pdx1, and insulin in the induced MSCs (Figures 1B,C), with approximately 80% of the induced MSCs showing positive for both insulin and Pdx1 and the remainder showing negative for these islet hormones (Figure 1B). FCM confirmed the expression of Ngn3 and Pdx1 in the induced MSCs with no expression observed in the uninduced MSCs (Figure 1E). Insulin release is an important function of pancreatic beta cells, and GSIS as detected by ELISA demonstrated that the induced MSCs released insulin when treated with 5.5 and 20.5 mM glucose, while the uninduced MSCs did not release insulin *in vitro* (Figure 1D).

### The Expression of Specific Genes in the Five Stages of MSC Differentiation

**FIGURE 2:**
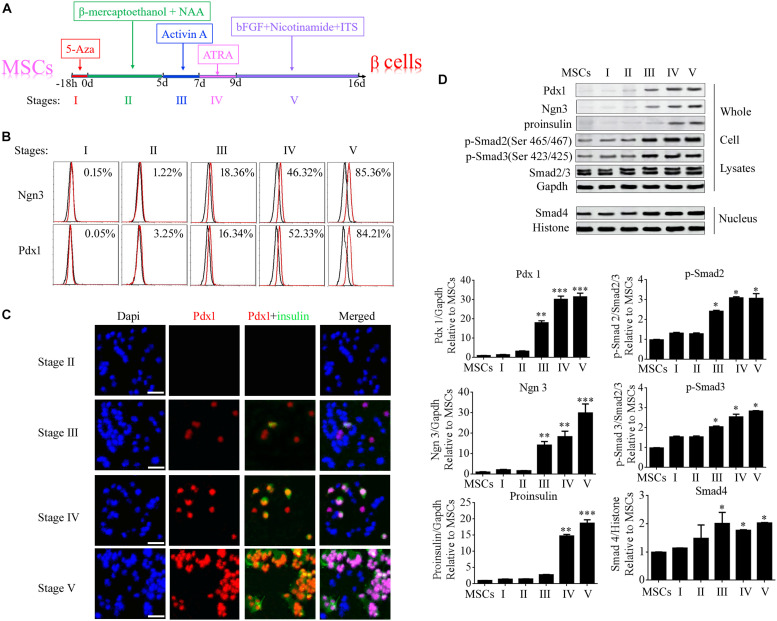
Specific genes were significantly up-regulated during stage III of beta cell differentiation from MSCs. **(A)** Schematic diagram of the differentiation program. The program was divided into five stages according to the order in which the induction factors were added. **(B)** Flow cytometry analysis of the expression of Ngn3 and Pdx1 during each stage of differentiation. **(C)** Immunofluorescence microscopy was used to determine the expression of Pdx1 and insulin during each stage of differentiation. **(D)** Western blotting was used to analyze the expression of proteins during each stage, including the islet hormones, Ngn3 and Pdx1, and the main members of the TGF-β/Smad pathway, namely p-Smad2, p-Smad3, and Smad4. The data showed that these proteins were markedly elevated from stage III, implying that the TGF-β/Smad pathway plays an important role in beta cell differentiation. Values represent the mean ± SEM, *n* = 3. The paired two-tailed *t*-test was used for comparisons (^∗^*p* < 0.05, ^∗∗^*p* < 0.01, ^∗∗∗^*p* < 0.001). Gapdh and Histone were used as endogenous controls in the lysate and nuclear fractions, respectively.

Differentiation of umbilical cord MSCs into beta cells was induced by treatment with 5-AZA, β-mercaptoethanol, non-essential amino acids, ATRA, TSA, and nicotinamide. To elucidate the molecular mechanisms of beta cell differentiation from MSCs, the differentiation program was divided into five stages (Figure 2A). Ngn3 (Neurogenin3) is a member of the bHLH (basic helix-loop-helix) family of transcription factors involved in the development of neural stem cells in the neuroectoderm, and it is expressed in scattered cells throughout the embryonic pancreas (Gradwohl et al., 2000). During the generation of the pancreas, Ngn3 is required for the development of pancreatic endocrine cells and it is considered a marker for islet precursor cells (Habener et al., 2005). The *Pdx1* (pancreatic and duodenal homeobox 1) gene is also known as *Ipf1* (insulin promoter factor 1) or *Idx1* (islet/duodenum homeobox 1), and it is selectively expressed in islet beta cells where it binds to the promoter region of the insulin gene in the mouse. Therefore, in the present study, the expression of Ngn3 and Pdx1 was determined at each stage of the differentiation program using FCM, immunofluorescence microscopy, and western blotting. The data showed that Ngn3 and Pdx1 were significantly elevated from stage III and insulin was elevated from stage IV (Figures 2B–D). Activin A is a member of the Activin family, which is a major branch of the TGF-β superfamily. In stage III, activin A was added to the MSCs to continue the induction of their differentiation into beta cells. The expression of the main members of the TGF-β superfamily, Smad2/3, Smad4, phosphorylated (p-)Smad2, and p-Smad3, was also determined using western blotting. The results demonstrated that cytoplasmic levels of p-Smad2 and p-Smad3 and the nuclear level of Smad4 were significantly up-regulated from Stage III following activin A treatment (Figure 2D). These data implied that the TGF-β/Smad pathway plays an important role in inducing the formation of beta cells from stem cells.

### Pancreatic miRNAs Also Exhibited Increased Expression From Stage III of MSC Differentiation

**FIGURE 3:**
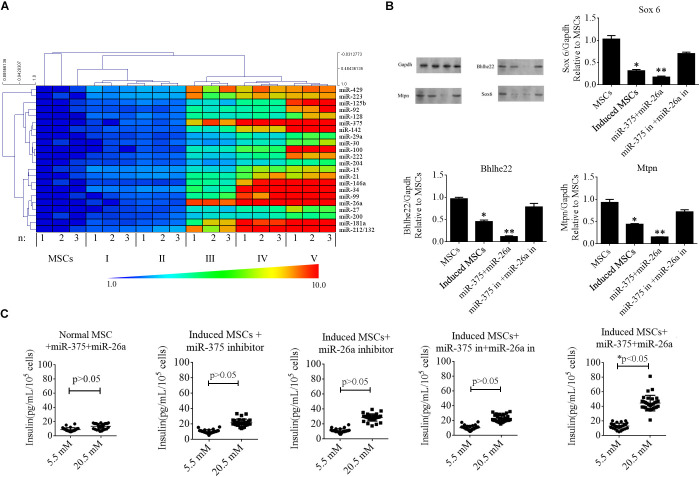
Roles of specific miRNAs in beta cell differentiation from MSCs. **(A)** A heat map was used to represent the fold change in the expression of specific miRNAs during the five stages of differentiation of beta cells from MSCs based on RT-qPCR. MiR-375 and miR-26a were among those exhibiting the greatest increase in expression. **(B)** Western blotting analysis of targets of miR-375 and miR-26a in induced MSCs following the overexpression of miR-375 and miR-26a or that of their inhibitors (in). Over-expression of miR-375 and miR-26a inhibited the endogenous expression of Sox 6, Bhlhe22, and Mtpn, while the over-expression of miR-375 and miR-26a inhibitors enhanced the expression of these targets. Gapdh was used as an endogenous control. Values represent the mean ± SEM, *n* = 3. **(C)** Glucose-stimulated insulin secretion (GSIS) analysis of induced MSCs following the overexpression of miR-375 and miR-26a or that of their inhibitors (in). The data show that insulin secretion of all groups of induced MSCs decreased when the cells were treated with a higher glucose concentration except when miR-375 and miR-26a were over-expressed (*n* = 30; paired two-tailed *t*-test).

MicroRNAs in mammals exhibit developmental stage-specific or tissue-specific expression, implying that miRNAs play important roles in development processes. Several miRNAs regulate the development of beta cells and their secretion of insulin, including miR-375, miR-124a, and miR-26a. In the present study, we reviewed previous reports to identify the miRNAs expressed in developing or adult pancreatic tissue and/or that play a functional role in islet or insulinoma cells (Supplementary Table S2). To determine whether these miRNAs were up-regulated in the induced MSCs, we performed RT-qPCR at every stage of beta cell differentiation. The data were analyzed with the 2^–ΔΔCT^ method and illustrated with a heat map (Figure 3A). The results indicated that specific miRNAs were dramatically elevated from stage III to V, with miR-375 and miR-26a being among those exhibiting the greatest increase in expression. The expression levels of miR-375 and miR-26a were positively correlated with those of Ngn3 and Pdx1 (determined in the previous section) for the normal MSCs and induced MSCs (*p* < 0.05). To determine whether the expression of miR-375 and miR-26a could induce the differentiation of beta cells from normal MSCs without cocktail factor treatment, we synthesized miR-375 and miR-26a mimics for co-transfection into normal MSCs, however, Pdx1 and proinsulin remained negative for expression after 16 days post-transfection (Supplementary Figure S1).

In our previous report (Bai et al., 2017a), miR-375 and miR-26a combined with niacinamide were shown to promote beta cell differentiation from MSCs. We, therefore, determined the protein levels of the targets of miR-375 and miR-26a, namely Sox6, Bhlhe22, and Mtpn, and found these proteins to be markedly down-regulated after cocktail factor treatment, while the opposite trend was observed when the expression of miR-375 and miR-26a was inhibited in induced MSCs (Figure 3B). We next investigated the role of miR-375 and miR-26a in insulin secretion in induced MSCs with the GSIS method. Our results demonstrated that when miR-375 or miR-26a were over-expressed in combination or singly in induced MSCs, the beta cells derived from these MSCs exhibited normal promotion of insulin secretion, while the single or combined expression of miR-26a and miR-375 or its inhibitors decreased insulin secretion under the treatment of 20.5 mM glucose (Figure 3C). These results indicated that the TGF-β/Smad pathway plays an important role in the expression of these miRNAs, but the mechanisms by which the TGF-β/Smad pathway regulate miRNA transcription to promote beta cell differentiation from MSCs remain poorly understood.

### TGF-β/Smad Pathway Enhances the Transcription of miR-375, miR-26a, and Ngn3, Thereby Promoting Beta Cell Differentiation in Stage III

**FIGURE 4:**
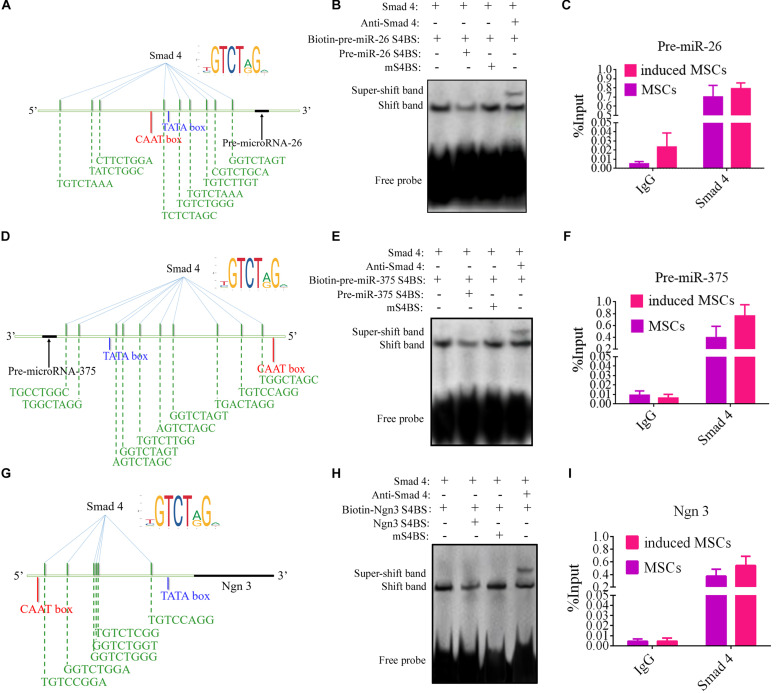
EMSA and ChIP-qPCR assay of the physical binding of Smad4 to the promoter regions of miR-375, miR-26a, and Ngn3. **(A**,**D**,**G)** Diagrams indicating the predicted binding sites for Smad4 within the promoters of miR-375, miR-26a, and Ngn3. **(B**,**E**,**H)** EMSA of the physical binding of Smad4 to the promoter regions of miR-375, miR-26a, and Ngn3. The promoter Smad4-binding sites with the highest scores based on bioinformatic predictions were selected for EMSA probe design. Following induction, cell nuclear extracts were prepared and incubated with the specific biotinylated EMSA probe for Smad4. Non-biotinylated EMSA probes were used as competitor probes. Smad4 was observed to bind to each of the three predicted binding sites incorporated into the EMSA probes. Furthermore, incubation with specific antibodies against Smad4 clearly resulted in supershifted bands. Probes containing mutated binding sites (mS4BS) were unable to compete for binding. S4BS, Smad4-binding site. **(C**,**F**,**I)** The amounts of Smad4 enriched within the promoter regions of miR-375, miR-26a, and Ngn3 in uninduced and induced MSCs were quantified by ChIP-coupled real-time qPCR. The percentages of input were calculated according to the threshold cycle values (CT). Non-specific IgG was used as the negative control. The amounts of immunoprecipitated chromatin were significantly higher for the induced MSCs than the uninduced MSCs, with a fold change in occupancy of 8.36, 7.89, and 6.61 for miR-375, miR-26a, and Ngn3, respectively. Values represent the mean ± SEM, *n* = 3.

Activin A is an activator of the TGF-β/Smad pathway. In earlier experiments, this protein was found to significantly elevate the levels of p-Smad2 and p-Smad3 in the whole-cell lysates of induced MSCs, as well as the nuclear expression of Smad4, from differentiation stage III onward. Phosphorylated Smad2 and Smad3 form a complex with Smad4 to translocate it into the nucleus, wherein it regulates the transcription of its target genes. To determine the mechanisms causing the elevated levels of specific genes from differentiation stage III, the ALGGEN-PROMO (version 8.3) (Farre et al., 2003) and JASPAR (version 7.0) (Khan et al., 2017, 2018) tools were used to screen for potential Smad4-binding sites in the promoter regions of these genes. PROMO is a classic bioinformatics tool for the selection of putative transcription factor-binding sites in promoter sequences of target genes and it makes use of the TRANSFAC database. Transcription factor-binding sites defined in the TRANSFAC database are used to construct specific binding site weight matrices for transcription factor-binding site prediction. JASPAR is an open-access database of curated, transcription factor binding profiles stored as position frequency matrices and transcription factor flexible models for transcription factors across multiple species in six taxonomic groups. By combining these tools, we found many putative Smad4-binding motifs within the promoter regions of miR-375, miR-26a, and Ngn3 but none were identified for Pdx1 (Figures 4A,D,G). Ngn3 is the earliest factor that specifically regulates the development of the endocrine compartment in the embryonic pancreas, and it regulates the transcription of many beta cell genes (Habener et al., 2005; Sancho et al., 2014).

Interactions between transcript factors and nucleic acids are usually tested using EMSA. In this study, to investigate the interactions predicted by bioinformatic analysis, probes for EMSA were designed for Smad4 based on the putative binding sites within the promoter sequences of its target genes with the highest probability score for Smad4 binding. The EMSA data demonstrated that Smad4 did indeed bind to the predicted binding sites (Figures 4B,E,H). The specificity of protein binding to these sites was further verified by competition with a 100–200-fold molar excess of unlabeled EMSA probe. Supershifted bands were also observed following incubation with the Smad4 antibody (ChIP grade). To accurately compare the quantities of Smad4 enriched in the promoter region after induction, ChIP was combined with qPCR analysis. The input sample (whole lysate) was used as a positive control, and non-specific IgG was used as a negative control. The quantities of immunoprecipitated chromatin were dramatically changed in the induced MSCs compared to the uninduced MSCs, with a fold change in occupancy of 8.36, 7.89, and 6.61 for miR-375, miR-26a, and Ngn3, respectively (Figures 4C,F,I).

**FIGURE 5:**
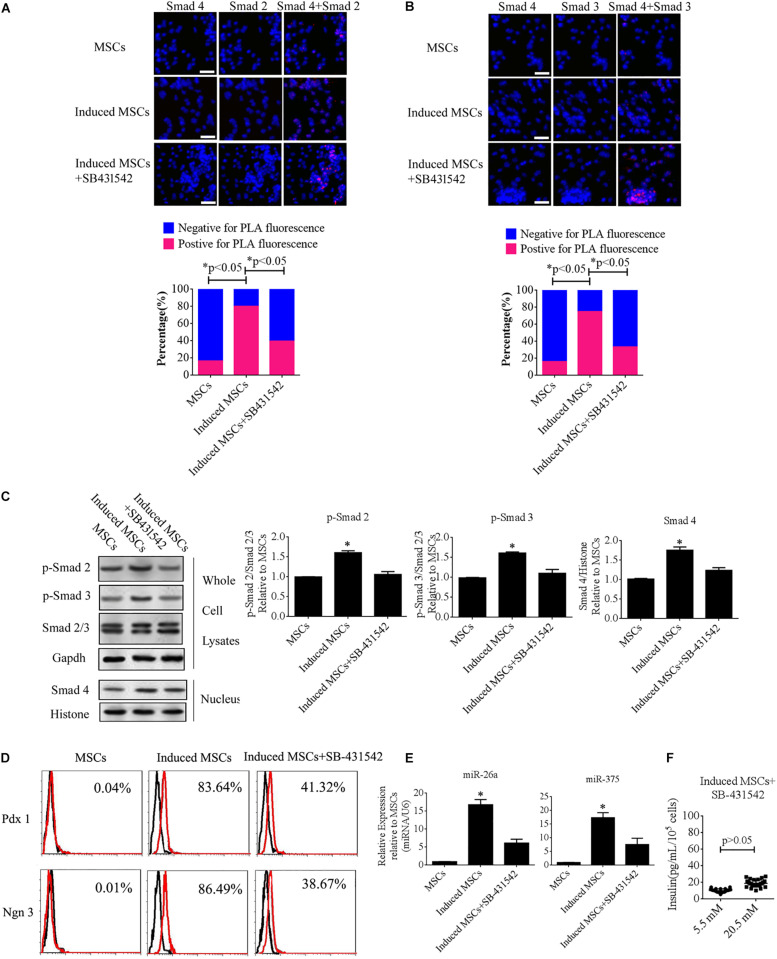
SB-431542 inhibits beta cell differentiation from MSCs by blocking the TGF-β/Smad pathway. **(A,B)** Duolink PLA fluorescence assay of the interactions of p-Smad2 or p-Smad3 with Smad4 in MSCs following different treatments. Colocalization of p-Smad2 or p-Smad3 with Smad4 around and within nuclear foci is shown in the upper panel, which corroborated the interaction of these proteins in uninduced and induced MSCs (scale bar = 50 μm). The percentages of PLA-positive cells following the different treatments are shown in the bottom panel. The number of positive cells was significantly elevated after treatment with the cocktail of factors compared with uninduced MSCs or SB-431542-treated induced MSCs. **(C)** Western blotting showed that SB-431542 down-regulated p-Smad2 and p-Smad3, which led to decreased levels of intranuclear Smad4. **(D)** Flow cytometry analysis of Pdx1 and Ngn3 expression showed that the percentage of Pdx1- and Ngn3-positive induced MSCs was markedly decreased after SB-431542 treatment. **(E)** RT-qPCR demonstrated that SB-431542 prevented activation of the TGF-β/Smad pathway leading to reduced expression of miR-375 and miR-26a. **(F)** Glucose-stimulated insulin secretion (GSIS) of induced MSCs after SB-431542 treatment indicated that insulin secretion under conditions of 20.5 mM glucose increased to a much smaller degree than that in cells not treated with the inhibitor (*n* = 30; paired two-tailed *t*-test).

To further illuminate the role of the TGF-β/Smad pathway in the differentiation of beta cells from MSCs, SB-431542 (a TGF-β/Smad pathway inhibitor) was added to the cocktail of factors. The levels of p-Smad2, p-Smad3, and intranuclear Smad4, as well as the interactions of Smad2 or Smad3 with Smad4, were analyzed using Duolink PLA and western blotting. The data showed that the number of PLA-positive cells was dramatically increased under treatment with the cocktail factors compared with the uninduced MSCs, while treatment with SB-43152 produced much fewer PLA-positive cells relative to the induced MSCs alone (Figures 5A,B). We then performed western blotting of SB-431542-treated induced MSCs using specific antibodies against p-Smad2, p-Smad3, and intranuclear Smad4. The results showed that SB-431542 down-regulated p-Smad2 and p-Smad3, which led to decreased levels of intranuclear Smad4 (Figures 5C). To test for beta cell differentiation after SB-431542 treatment, the percentage of Pdx1- and Ngn3-positive cells, the expression levels of miR-375 and miR-26a, and the level of insulin secretion after treatment were verified using FCM, qPCR, and the GSIS method, respectively. The results showed that all three measures were dramatically decreased after SB-431542 treatment (Figures 5D–F). These data confirmed that the TGF-β/Smad pathway plays an important role in beta cell differentiation.

### Transplantation *in vivo*

**FIGURE 6:**
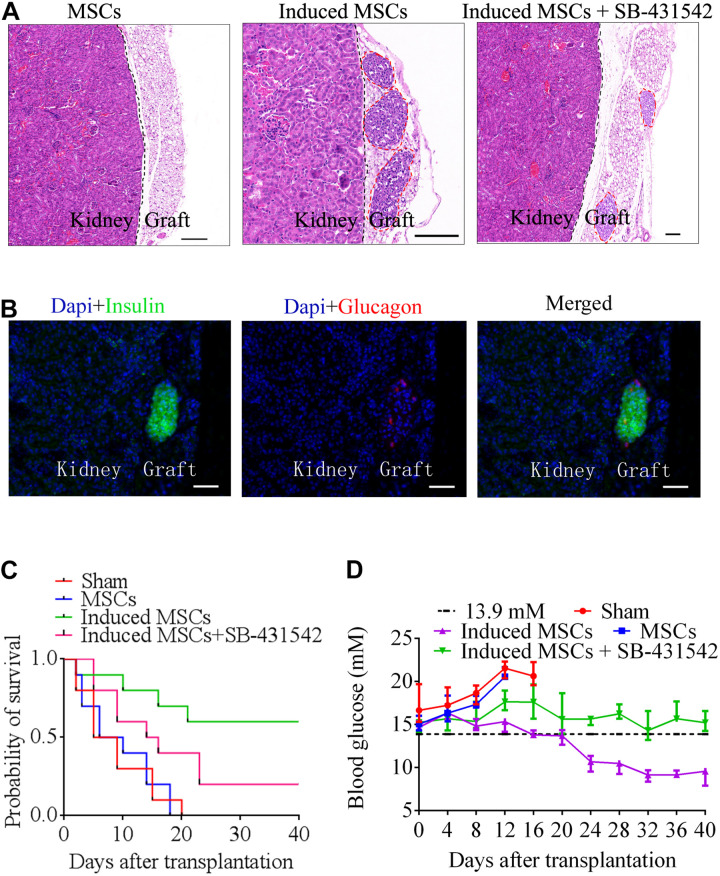
Beta cells differentiated from MSCs were transplanted below the renal capsule of STZ-treated diabetic mice. **(A)** Hematoxylin and eosin staining of a graft at day 40 after transplantation. Higher numbers of islet-like structures were observed in induced MSCs than in SB-431542-treated induced MSCs (red circles). The MSC-transplanted group did not exhibit any of these structures in the graft 18 days after transplantation. **(B)** Immunofluorescence images of a graft at day 40 after transplantation, stained for insulin and glucagon to confirm the presence of engrafted islets (scale bar = 50 μm). **(C)** Survival curves of diabetic mice following transplantation. Induced MSC-transplanted diabetic mice (*n* = 10) survived the longest with a survival probability reaching 60%, while the survival probability of SB-431542-treated induced MSC-transplanted diabetic mice (*n* = 10) only reached 20%, and that of the sham control and MSC-transplanted groups (*n* = 10) was 0%. **(D)** Blood glucose analysis of the various transplantation groups showed that the induced MSC-transplanted diabetic mice recovered to normal glucose levels (<13.9 mM) after 20 days. The other groups retained high levels of blood glucose (>13.9 mM) over the 40 days observation period.

To confirm the *in vivo* function of beta cells differentiated from MSCs, we transplanted MSCs, induced MSCs, and SB-431542-treated induced MSCs under the kidney capsule of STZ-treated diabetic mice (*n* = 10). After transplantation, the sham group and MSC-transplanted mice all died within 16 and 20 days due to persistent metabolic acidosis, respectively. The survival probability of induced MSC-transplanted mice was approximately three times higher than that of SB-431542-treated induced MSC-transplanted mice (Figure 6C). Twenty days after transplantation, the blood glucose of the induced MSC-transplanted mice decreased to a normal level (less than 13.9 mM). In contrast, the blood glucose of the SB-431542-treated induced MSC-transplanted mice remained above 13.9 mM (Figure 6D). At 40 days post-transplantation, the mice were sacrificed and the engrafted kidneys underwent histologic analysis. Hematoxylin and eosin staining revealed the presence of pancreatic beta cells adjacent to the mice kidney in the induced MSC-transplanted mice and SB-431542-treated induced MSC-transplanted mice, while none were observed in the MSC-transplanted mice (Figure 6A). IHC showed the presence of pancreas islet-like structures adjacent to the mouse kidney (Figures 6B). These data revealed that the induced MSCs, when transplanted into the renal capsule, could improve blood glucose control and functionally rescue the STZ-treated diabetic mice, while SB-431542 treatment of the induced MSCs did not have this beneficial effect.

**FIGURE 7:**
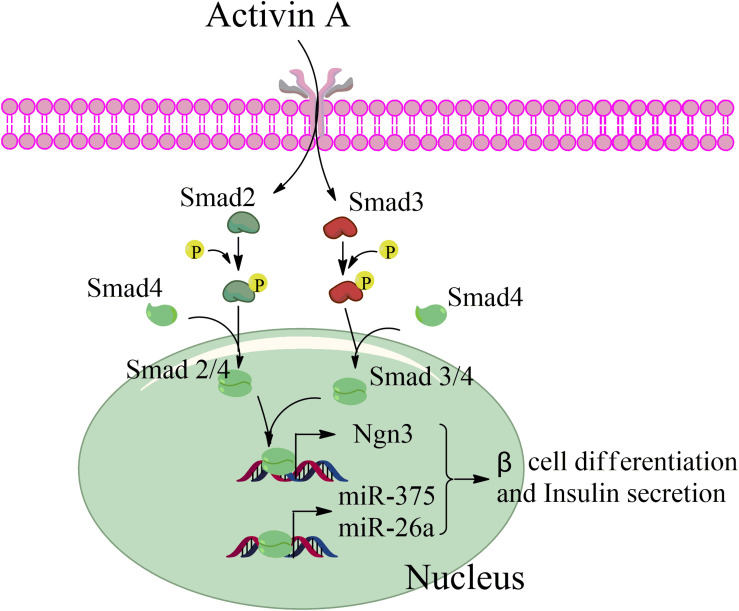
Schematic of the factors promoting pancreatic beta differentiation from mesenchymal stem cells.

Taken together, our data revealed that activin A activates the TGF-β/Smad pathway through phosphorylation of Smad2 and Smad3 during beta cell differentiation from MSCs, and that phosphorylated Smad2 and Smad3 then interacts with Smad4 to form a transcription complex that enters the nucleus. This complex combines with the promotor sequences of the endocrine-specific transcription factor, Ngn3, and the pancreas-specific miRNAs, miR-375, and miR-26a, to enhance their transcription in stage III and thereby initiate MSC differentiation into beta cells (Figure 7).

## Discussion

The TGF-β superfamily is a component of a critical pathway involved in cell-to-cell signaling during a multitude of processes, including cell proliferation in somatic tissues, specification of cell fate during embryogenesis, cell differentiation, and cell death. Apart from TGF-β, the superfamily also includes the activins and the bone morphogenetic proteins (BMPs). In particular, activin A phosphorylates Smad2 and Smad3 to activate the TGF-β/Smad pathway, and this activin has been extensively used in the production of beta cells from stem cells (Shi et al., 2005; Pagliuca et al., 2014; Bai et al., 2017a) as it is important for early definitive endoderm development. Activin A binds to cell surface receptors to induce the expression of specific targets, including mix11 and goosecoid, thereby regulating the differentiation of pancreatic beta cells. In addition, activin A also promotes insulin secretion to maintain its function in cultured pancreatic islets (Florio et al., 2000; Li et al., 2004; Shi et al., 2005). However, there have been no reports to date regarding the mechanism by which activin A directly regulates the expression of pancreatic genes.

The endocrine-specific transcription factor, Ngn3, plays an important role in the production of beta cells from other pancreatic cells through cell reprogramming, including exocrine cells (Zhou et al., 2008) and ductal cells (Sancho et al., 2014). In pancreatic exocrine cells in mice, Ngn3 works in concert with two other transcription factors, Pdx1 and Mafa, to reprogram this differentiation to form cells that are indistinguishable from endogenous islet beta cells in size, shape, and ultrastructure. The ubiquitin ligase, Fbxw7, normally destabilizes Ngn3; genetic deletion of *Fbxw7* in pancreatic ductal cells, a type of stem cell in the pancreas, stimulates the direct conversion of these cells into endocrine β-cells (Sancho et al., 2014; Seifert and Xiong, 2014). In the present study, activin A was added into the differentiation program of beta cells in stage III, which activated the TGF-β/Smad pathway. Our results revealed that activin A enhanced the transcription of *Ngn3* through Smad4 binding of the promoter region of *Ngn3*. Therefore, activin A plays an important role in the transcription of endocrine-specific genes during beta cell differentiation from MSCs.

MicroRNAs play an important role in the regulation of gene expression at the post-transcriptional level (Bartel, 2004; Tang et al., 2007, 2009; Bai et al., 2017b). Many studies have investigated the expression and function of miRNAs in the formation of pancreatic beta cells from stem cells, but studies on the transcription of specific miRNAs during the formation of pancreatic beta cells from stem cells are limited. MiR-375 is a pancreas-specific miRNA that acts in endocrine tissues and is highly expressed in the pancreas (Poy et al., 2004; Avnit-Sagi et al., 2009; Bai et al., 2017a). Knockout of miR-375 disrupts islet morphogenesis and reduces endocrine cell differentiation. MiR-375 can induce the formation of pancreatic beta cells from adult or embryonic stem cells. Furthermore, miR-375 can also directly induce the differentiation of pancreatic beta cells from induced pluripotent stem cells (Poy et al., 2009; Bai et al., 2017a), however, the mechanism underlying this process remains unclear (Lahmy et al., 2014). MiR-26a was shown to be highly enriched in pancreatic beta cells in the current study, and it is known to be upregulated in response to glucose-stimulated repression of Sox6 and Bhlhe22 expression (Melkman-Zehavi et al., 2011). Furthermore, miR-26a can promote pancreatic cell differentiation both *in vitro* and *in vivo* by targeting the TET family of proteins (Fu et al., 2013). In our previous research, miR-375 and miR-26a were found to be dramatically elevated following the differentiation of pancreatic beta cells from MSCs (Bai et al., 2017a). The functions of miR-375 and miR-26a during this process lie not only in the promotion of differentiation but also in their effect on insulin secretion under glucose stimulation. However, the upstream mechanisms by which cocktail factors elevate the expression of miR-375 and miR-26a to promote the differentiation of pancreatic beta cells remain to be elucidated. In the present study, to investigate the expression of pancreatic-specific miRNAs during the differentiation of pancreatic beta cells from MSCs, we divided the differentiation program into five stages and tested the expression of specific miRNAs at each stage using RT-qPCR. The data showed that these miRNAs, particularly miR-375 and miR-26a, exhibited increased expression from stage III. We further demonstrated that the promoter regions of miR-375 and miR-26a were bound by activin A-activated Smad4, which enhanced their transcription and thereby promoted the differentiation of pancreatic beta cells from MSCs and increased their insulin secretion.

## Conclusion

In conclusion, we systematically elucidated the important role of activin A in inducing the differentiation of pancreatic beta cells from MSCs. Activin A phosphorylates Smad2 and Smad3 to activate the TGF-β/Smad pathway, thereby enhancing the transcription of the endocrine-specific transcription factor, Ngn3, which, in turn, promotes the differentiation of pancreatic beta cells. Additionally, miR-26a and miR-375, important miRNAs in the formation of beta cells and in modulating their secretion of insulin, are up-regulated by Smad4. *In vivo* and *in vitro* addition of an inhibitor of the TGF-β/Smad pathway, SB-431542, during the differentiation of the beta cells further demonstrated the critical role of the TGF-β/Smad pathway in the differentiation of beta cells and in insulin secretion. Our study, which particularly focused on the transcription of specific genes involved in beta cell formation from stem cells, may assist in the future development of effective cell transplant therapies for the treatment of type I diabetes mellitus.

## Data Availability Statement

The raw data used to support the conclusions of this article will be made available by the corresponding authors, without undue reservation, to any qualified researcher.

## Ethics Statement

The animal study was reviewed and approved by the Institutional Animal Care and Use Committee of Jining Medical University.

## Author Contributions

YG performed the cell differentiation, western blotting, and FCM, and drafted the manuscript. CB, SD, XZ, and RZ prepared the cell cultures. CB performed the RNAi assays. XL and CB analyzed the data and reviewed the manuscript. All authors contributed to manuscript revision, read and approved the submitted version.

## Conflict of Interest

The authors declare that the research was conducted in the absence of any commercial or financial relationships that could be construed as a potential conflict of interest.

## Footnotes

1 http://mev.tm4.org/
