# Corrigendum: Role of TGF-β/Smad Pathway in the Transcription of Pancreas-Specific Genes During Beta Cell Differentiation

**DOI:** 10.3389/fcell.2020.614840

**Published:** 2020-11-06

**Authors:** Yuhua Gao, Ranxi Zhang, Shanshan Dai, Xue Zhang, Xiangchen Li, Chunyu Bai

**Affiliations:** ^1^Institute of Precision Medicine, Jining Medical University, Jining, China; ^2^Institute of Animal Sciences, Chinese Academy of Agricultural Sciences, Beijing, China; ^3^Department of Spine Surgery, Qingdao Municipal Hospital, Qingdao, China; ^4^College of Animal Science and Technology, College of Veterinary Medicine, Zhejiang A&F University, Lin'an, China

**Keywords:** pancreatic beta cells, stem cells, TGF-β/Smad pathway, Ngn3, microRNAs

In the original article, there was a mistake in Figure 3B as published. The immunoblot bands of Mtpn was lost. The corrected Figure 3B appears below.

**Figure 3:**
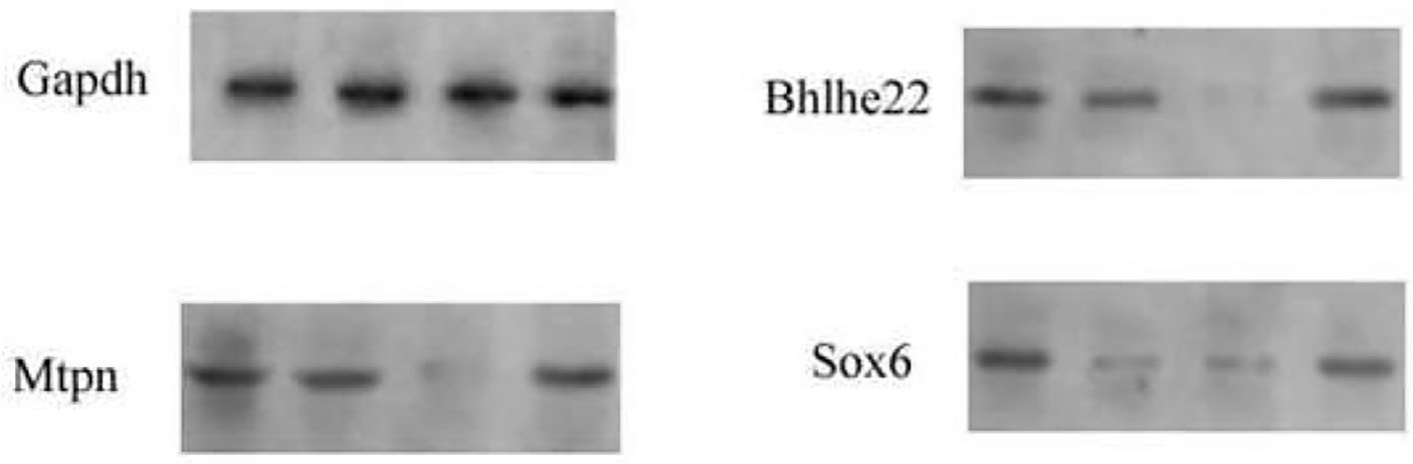
Western blotting analysis of targets of miR-375 and miR-26a in induced MSCs following the overexpression of miR-375 and miR-26a or that of their inhibitors (in).

The authors apologize for this error and state that this does not change the scientific conclusions of the article in any way. The original article has been updated.

