# Supplementary material for: Role of TGF-β/Smad Pathway in the Transcription of Pancreas-Specific Genes During Beta Cell Differentiation
Source: Front Cell Dev Biol. 2019 Dec 20;7:351. doi: 10.3389/fcell.2019.00351 (PMC6933421; doi:10.3389/fcell.2019.00351)
Supplement: Supplementary file 1 [file Data_Sheet_1.docx]

Supplementary Material


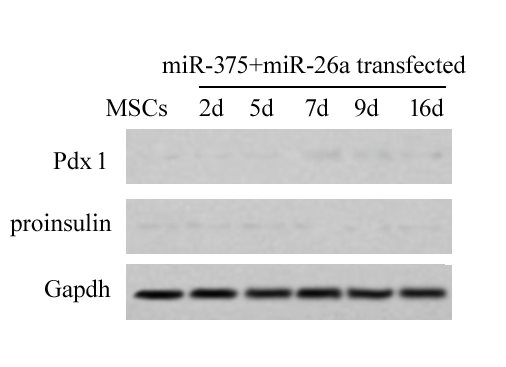


Figure S1 Negative expression of Pdx 1 and proinsulin after over-expressed miR-375 and miR-26a in MSCs.
