# Supplementary material for: Role of TGF-β/Smad Pathway in the Transcription of Pancreas-Specific Genes During Beta Cell Differentiation
Source: Front Cell Dev Biol. 2019 Dec 20;7:351. doi: 10.3389/fcell.2019.00351 (PMC6933421; doi:10.3389/fcell.2019.00351)
Supplement: Supplementary file 2 [file Table_1.docx]

Supplementary Material

Table S1 Probe used in Figure 4

| Probe | Probe sequence | |
| --- | --- | --- |
| Pro-miR-26a |  |  |
| Biotin-lable Probe | F | 5' CCCCGTCCCCCATCCTGTCTGGGCTCCAGGCTGGAGTCTG 3' |
|  | R | 5' CAGACTCCAGCCTGGAGCCCAGACAGGATGGGGGACGGGG 3' |
| non-lable Probe | F | 5' CCCCGTCCCCCATCCTGTCTGGGCTCCAGGCTGGAGTCTG 3' |
|  | R | 5' CAGACTCCAGCCTGGAGCCCAGACAGGATGGGGGACGGGG 3' |
| mutation Probe | F | 5' CCCCGTCCCCCATCCCTCACTGGCTCCAGGCTGGAGTCTG 3' |
|  | R | 5' CAGACTCCAGCCTGGAGCCAGTGAGGGATGGGGGACGGGG 3' |
| Pro-miR-375 |  |  |
| Biotin-lable Probe | F | 5' CCCTTTCTCTTTTGCAGGACAGACAACCTCTTGCAGATG 3' |
|  | R | 5' CATCTGCAAGAGGAAGTCTGTCCTGCAAAAGAGAAAGGG 3' |
| non-lable Probe | F | 5' CCCTTTCTCTTTTGCAGGACAGACAACCTCTTGCAGATG 3' |
|  | R | 5' CATCTGCAAGAGGAAGTCTGTCCTGCAAAAGAGAAAGGG 3' |
| mutation Probe | F | 5' CCCTTTCTCTTTTGCAGGATGACTGACCTCTTGCAGATG 3' |
|  | R | 5' CATCTGCAAGAGGAAGCAGTCACTGCAAAAGAGAAAGGG 3' |
| Ngn3 |  |  |
| Biotin-lable Probe | F | 5' TCCTTCATGCTACCAAGAAAGGGTCTGGACACATGCCAA 3' |
|  | R | 5' TTGGCATGTGTCCAGACCCTTTCTTGGTAGCATGAAGGA 3' |
| non-lable Probe | F | 5' TCCTTCATGCTACCAAGAAAGGGTCTGGACACATGCCAA 3' |
|  | R | 5' TTGGCATGTGTCCAGACCCTTTCTTGGTAGCATGAAGGA 3' |
| mutation Probe | F | 5' TCCTTCATGCTACCAAGAAAGAAGTGAGACACATGCCAA 3' |
|  | R | 5' TTGGCATGTGTCTGACTTCTTTCTTGGTAGCATGAAGGA 3' |

Table S2 Specific miRNAs involved in the regulation of pancreas development and insulin release

| microRNA name | Gene Functions |
| --- | --- |
| miR-26a | Regulates pancreatic cell differentiation via targeting TET family proteins, Sox6 and bHLHe 22 (Fu et al., 2013;Bai et al., 2017a). |
| miR-375 | Negative regulator of glucose-induced insulin secretion through targeting Mtpn (Poy et al., 2004;Bai et al., 2017a). miR-375 maintains normal pancreatic alpha cells, less beta cells mass (Poy et al., 2009). Regulation of PI3 pathway by regulation of PDK1 in insulinoma cells (Lim et al., 2005). The miR-375 promoter directs expression selectively to endocrine pancreas (Lewis et al., 2003). miR-375 promotes differentiation of human iPS cells into beta cell (Lahmy et al., 2014). |
| miR-100, miR-99 | miR-100 is a non-specific microRNA in pancreas, but it can promote increased cell proliferation and a reduction in apoptosis (Morais et al., 2014). |
| miR-125b | Nestin is a target of miR-125b; it down-regulates the expression of Nestin protein (Chabot et al., 2015). |
| miR-181a | Regulates expression of Sirtuin-1 in hepatocytes for improving insulin sensitivity (Zhou et al., 2012). A regulator of pancreatic cancer invasion and progression (Liu et al., 2014). |
| miR-92 | Over-expression of miR-92 changes the level of intracellular ions (Barbato et al., 2010). |
| miR-30 | miR-30 family miRNAs form part of the regulatory signaling events involved in the cellular response of pancreatic epithelial cells during mesenchymal transition (Joglekar et al., 2009). |
| miR-27 | Regulates fat metabolism (Lin et al., 2009), osteoblast differentiation (Wang and Xu, 2010) and cardiogenesis (Chinchilla et al., 2011). |
| niR-142 | Inhibits Pancreatic ductal adenocarcinoma cell growth *in vitro* and blocked metastatic spread i*n vivo* (MacKenzie et al., 2013). |
| miR-429 | Modifies the hypothalamo-pituitary-ovarian axis to promote ovulation in mouse (Hasuwa et al., 2013). |
| miR-29a, miR-222 | Has a crucial role in cell proliferation and development of pancreatic cancer via targeting the Ki67 gene (Lee et al., 2013). miR-222 is also a potential regulator of ERalpha expression in estrogen-induced insulin resistance in gestational diabetes mellitus and might be a candidate biomarker and therapeutic target for gestational diabetes mellitus (Shi et al., 2014). |
| miR-200, miR-128,  miR-204, miR-223 | Modulates aggressive behavior in pancreatic cancer cells and other cancer cells (Soubani et al., 2012;Woo et al., 2012;Chen et al., 2013;Ma et al., 2013). |
| miR-146a | Increases expression in islets from db/db obese mice, contributes to fatty acid-induced beta-cell dysfunction. Pro-inflammatory cytokines induce its expression in human islet and MIN6 cells (Huang et al., 2007). |
| miR-15 | Role in pancreatic regeneration, possibly by targeting Ngn3 (Joglekar et al., 2007). |
| miR-21 | Pro-inflammatory cytokines induce its expression in human islet and MIN6 cells (Huang et al., 2007). Acts as a bidirectional switch in the formation of IPCs by regulating the expression of SOX6, RPBJ and HES1. |
| miR-34 | miR-34 contributes to fatty acid-induced beta cell dysfunction (Rosero et al., 2010). miR-34c was shown to downregulate PDE7B, PDGFRA, and MAP2K1 to increase proinsulin synthesis. |
| miR-212/132 | miR-212/132 enhances glucose and GLP-1 stimulated insulin secretion (Shang et al., 2015) |
